# Effectiveness of glyceryl trinitrate (GTN) vasodilator patches in peripheral arterial disease

**DOI:** 10.1186/1757-1146-8-S2-P8

**Published:** 2015-09-22

**Authors:** Sylvia McAra

**Affiliations:** 1 School of Community Health, Charles Sturt University, Thurgoona, NSW, 2640, Australia

**Keywords:** glyceryl trinitrate, toe pressure, vasodilation, ischemia

## Background

Foot ulceration is caused by a variety of factors including ischemia. GTN is a nitric oxide donor that reliably causes vasodilation, but its potential to improve local vascular supply to at-risk feet has received little attention.

When used to supplement standard evidence-based wound care, transdermal GTN produced rapid ulcer healing in four cases of non diabetic and diabetic ulcers. Subsequently, a larger study was conducted to investigate the effect of using small doses of GTN for therapeutic neurological and vascula outcomes in people with subnormal toe pressures.

## Methods

100 participants with toe brachial pressure indices (TBPIs) < 0.65 were allocated to four groups: two with low doses of GTN patch medication, a placebo group, and a control group. GTN doses were 1.25 mg and 2.5 mg applied constantly over 24 hours and replaced daily. Only the foot with the lower toe pressure received GTN treatment. Participants were assessed on both feet monthly for 6 months post intervention. High TBPI variability was noted in 14 participants, most of whom had unstable hypertension which was an exclusion criterion. Analysis proceeded with the remaining 86.

## Results

At 1 month post intervention, the TBPIs of the high dose group were significantly higher than those of the control group, *p* = 0.012. At 6 months post intervention, the TBIs of both the high dose and the low dose groups were significantly higher than those of the control group, with *p*= 0.044 and 0.048 respectively. The placebo group's TBIs were also higher than those of the control group, *p*= 0.006.

## Conclusions

GTN may increase vascular toe perfusion and thereby assist in wound care and prophylaxis, and might be considered as an adjunct to other therapy, particularly when wounds fail to heal. Unexpectedly high placebo group responses require repeat investigation.

